# Structural and Physical Characteristics of Mixed-Component Oleogels: Natural Wax and Monoglyceride Interactions in Different Edible Oils

**DOI:** 10.3390/gels9080627

**Published:** 2023-08-04

**Authors:** Dafni Dimakopoulou-Papazoglou, Foteini Giannakaki, Eugenios Katsanidis

**Affiliations:** Department of Food Science and Technology, School of Agriculture, Faculty of Agriculture, Forestry and Natural Environment, Aristotle University of Thessaloniki, 54124 Thessaloniki, Greece; ddimakop@agro.auth.gr (D.D.-P.); fgiannak@agro.auth.gr (F.G.)

**Keywords:** oleogels, waxes, monoglycerides, edible oils, lipid structuring, FTIR

## Abstract

Waxes and monoglycerides (MGs) added in edible oils form oleogels that can be used as an alternative structured fat, providing healthier substitutes to saturated and *trans* fats in foods. This study aimed to investigate the properties of oleogels formed by the interaction between monoglycerides and different waxes in various edible oils. For this purpose, waxes, namely rice bran (RBW), candelilla (CDW), sunflower (SW), and beeswax (BW), together with MGs in a total concentration level of 15% (*w*/*w*) were dissolved in several edible oils (olive, sunflower, sesame, and soybean). The structure and physical properties of oleogels were investigated using texture analysis, polarized light microscopy, melting point measurements, and Fourier-transform infrared spectroscopy (FTIR). The hardest structure was produced by SW/MG (5.18 N), followed by CDW (2.87 N), RBW (2.34 N), BW (2.24 N) and plain MG (1.92 N). Furthermore, RBW and SW led to a higher melting point (69.2 and 67.3 °C) than the plain MG oleogels (64.5 °C). Different crystallization structures, i.e., needle-like crystals and spherulites, were observed depending on the type of wax, its concentration, and the oil used. These results can be used to control the properties of oleogels by adjusting the gelator composition for a variety of potential food applications.

## 1. Introduction

Traditionally, solid and semisolid fat, used to formulate food products in the food industry, is structured by crystalline triacylglycerols (TAGs), which mainly consist of saturated fatty acids (SFAs) and may contain significant amounts of *trans* fatty acids (tFAs). However, the consumption of these types of fatty acids has been linked to an increase in risk for several diseases, with cardiovascular disease being the predominant one [1,2,3]. Therefore, the Food and Agriculture Organization of the United Nations (FAO) and the World Health Organization (WHO) have suggested a reduction in the intake of SFAs and tFAs at 10% and 1% of total energy intake, respectively [1,4]. Considering these regulations and changing consumer preferences, alternative methods are being investigated for partial or total replacement of SFAs in food products in order to improve their nutritional profile.

An alternative technology of reducing SFA and tFA content is structuring liquid edible oils, known as oleogelation, which has piqued the interest of the food industry in recent years [5,6]. Oleogelation is a process where a small amount of one or more gelator molecules are added to entrap liquid vegetable oils into a three-dimensional network to create structures with similar properties to conventional fat [7,8,9,10,11]. The properties of the oleogels are influenced by the type of gelator, the oil used, as well as the environmental and processing conditions [3]. Various gelators have been used for creating oleogels, including monoglycerides (MGs), fatty acids and fatty alcohols, lecithin, sterols, waxes, and other surfactants [8,12].

Among the various gelators, MGs have been extensively studied due to their ability to form networks of crystalline particles similar to those of hardstock fats [13]. The properties of MG oleogels, such as hardness, stability, elasticity, etc., render them suitable for many different food applications [14,15,16]. As pointed out in the literature, the structure and the physical properties of the MG oleogels are affected by the type and the concentration of the MGs, the oil type, and the processing conditions, namely temperature, stirring speed, cooling rate, storage temperature and time, etc., as well as the presence of other gelators [13]. Some studies have recently reported the interaction of MGs with other structuring agents, such as phytosterols [17,18,19], ethyl cellulose [20,21], and waxes [22,23,24], etc., so that oleogels can be more stable and with more suitable properties, depending on the intended food use.

Waxes are another type of ingredient utilized as gelling agents, owing to their ability to create a well-defined crystalline network with potent oil-binding properties [5], and they can be effective even at low concentrations [6]. The gelling behavior of waxes in edible oils is highly affected by the crystal morphology (namely, the rodlike or platelet crystals tend to form stronger gels), which in turn is governed by the chemical components present in waxes, and specifically their chain length [25]. Generally, waxes consist of various chemical components including straight-chain alkanes, long-chain fatty acids, long-chain fatty alcohols, and wax esters [5]. Several studies have documented the way in which natural waxes, such as beeswax [26,27], carnauba wax [28,29], candelilla wax [30,31], rice bran wax [6,32], or sunflower wax [33,34,35] crystallize in low concentrations when combined with liquid vegetable oils, such as sunflower, canola, or soybean oil. Also, different wax combinations [36,37,38] and candelilla wax–MG [22] and beeswax–ethyl cellulose [39] combinations have been studied. However, these studies mainly investigated the use of low concentrations of waxes, at which very soft gels are formed. For animal fat substitution, much harder materials and higher concentrations of structurants are typically required. To the best of our knowledge, studies aimed at the formation of shelf-stable gels based on the combination of waxes and MGs for the possible replacement of animal fat in foods, utilizing olive or sesame oil, are scarce and not comprehensive. Employing waxes to create structured oil is a promising proposition for the food industry, as the majority of waxes are food-grade and readily available at a low cost [5].

In view of the above, the aim of the present study was twofold: on the one hand, to design and study the properties of oleogels structured with waxes in combination with MGs for their potential application in fat replacement in foods, and on the other hand to reduce the amount of MG, maintaining the structural stability of the gels. For this purpose, a comprehensive study to simultaneously assess the impact and the interactions of different waxes and different oils on the structural properties of the resulting oleogels was designed. Specifically, four waxes of plant and animal origin, namely rice bran (RBW), candelilla (CDW), sunflower (SW), and beeswax (BW), both alone or together with MGs in a total concentration level of 15% (*w*/*w*) were dissolved in different edible oils, i.e., olive, sunflower, sesame, and soybean oil. The physical properties of the produced oleogels were studied, assessing the crystal morphology, texture properties, and melting behavior. The purpose of utilizing various edible oils in this study was to evaluate the influence of the type of liquid oil, namely, the different content of fatty acids, the degree of saturation/unsaturation, and the different content of other compounds, on the gelation and physical properties of oleogels.

## 2. Results and Discussion

### 2.1. Oleogel Appearance

**Figure 1:**
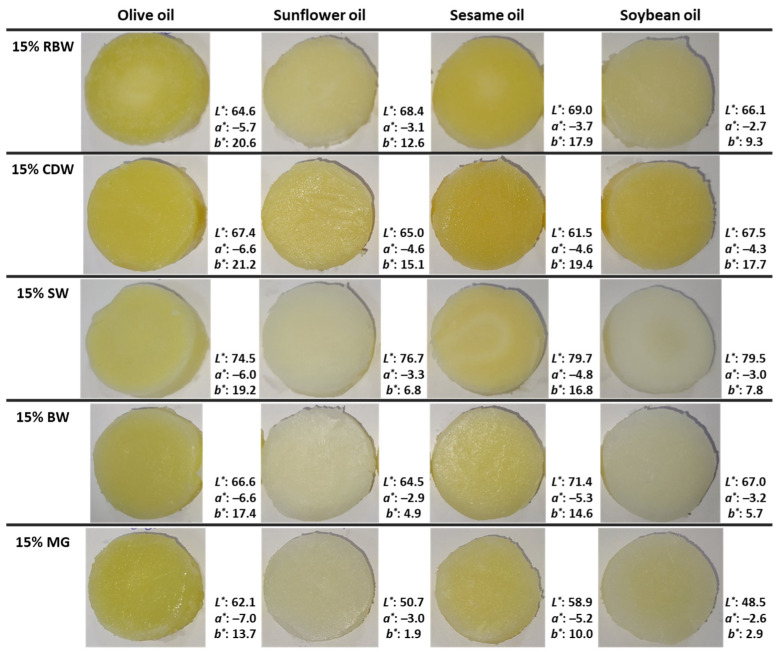
Optical images and color parameters (L*, a*, and b*) of oleogels prepared with 15% (*w*/*w*) rice bran wax (RBW), candelilla wax (CDW), sunflower wax (SW), beeswax (BW), and monoglycerides (MGs) in different edible oils.

The appearance and color values of oleogels created by a single structurant, i.e., a wax or MG in different edible oils are depicted in Figure 1. The use of different structural agents and different edible oils had a significant impact on the color of oleogels. In Table 1, the results of the full-factorial statistical analysis are presented. The average values for the instrumental color parameters L*, a*, and b* are presented for each main factor (e.g., structurant concentration, wax concentration, and edible oil type). Regarding the L* parameter, the SW oleogels had the highest values in all oils used (Table 1), indicating the highest lightness. On the contrary, MG oleogels had the darkest color in all examined oils compared to wax-based oleogels. It is noted that as the amount of MG increased in the oleogels, the L* values decreased, and, therefore, the lightness of the oleogel color decreased (Table 1). Regarding the different edible oils, sesame oil and olive oil wax-based oleogels achieved higher L* values than oleogels formed by sunflower oil and soybean oil. This indicates that the color of the edible oil significantly affects the final color of the oleogels (sunflower oil and soybean oil have a lighter color than sesame oil and olive oil). Parameter a* had negative values in all oleogels (Figure 1), indicating a green hue. Comparing the waxes used for oleogel structuring, CDW oleogels had the lowest a* value, while RBW oleogels resulted in the highest. Regarding the different edible oils, oleogels created by olive oil had the lowest a* value (a*: −6.7), followed by sesame oil (a*: −4.9), while oleogels formed by sunflower oil (a*: −3.5) and soybean oil (a*: −3.4) had the highest a* values. This is due to the color of the oil; specifically, olive oil has a greener hue than the other oils, affecting the final color of the oleogels. On the contrary, sunflower oil and soybean oil are refined and therefore have a lighter color which affects to a lesser degree the parameter a* of the oleogels. All oleogels had positive b* values, indicating a yellow hue. For all structural agents, the use of olive oil led to a greater intensity of yellow color (b*: 16.1), followed by sesame oil (b*: 13.9). In contrast, sunflower oil and soybean oil led to significantly less yellow oleogels (b*: 5.7 and 5.8, respectively), as illustrated in Figure 1, which is directly related to the color of the oils. The yellow hue of the oleogels is affected not only by the color of the edible oil used, but also by the structural agent dissolved in it. Overall, CDW formed the yellowest oleogels (b*: 13.9), followed by RBW (b*: 12.3), SW (b*: 10.5), BW (b*: 7.9), and MG (b*: 7.1). 

### 2.2. Polarized Microscopy

**Figure 2:**
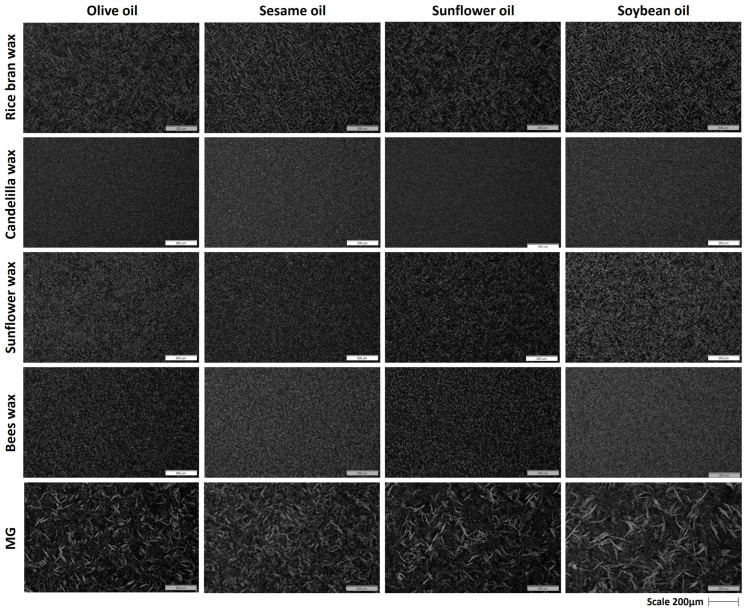
Polarized light micrographs of oleogels prepared with 15% (*w*/*w*) rice bran wax, candelilla wax, sunflower wax, beeswax, and monoglycerides in different edible oils (scale 200 μm).

**Figure 3:**
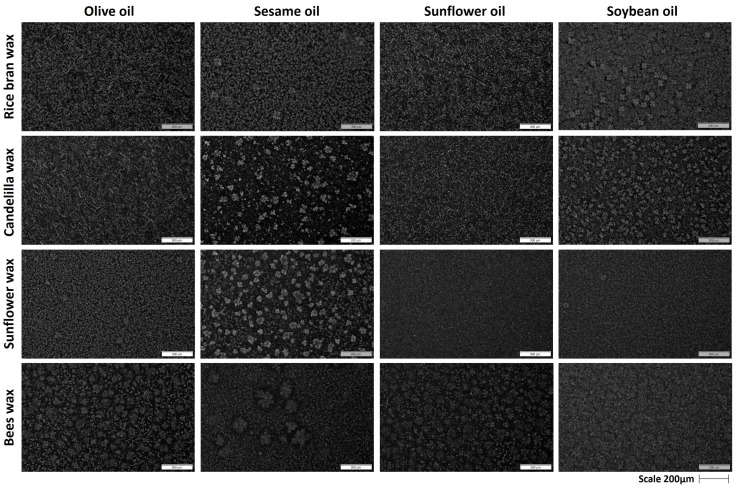
Polarized light micrographs of oleogels prepared with 7.5% (*w*/*w*) monoglycerides combined with 7.5% (*w*/*w*) rice bran wax, candelilla wax, sunflower wax, or beeswax, in different edible oils (scale 200 μm).

Micrographs of oleogels created by a single structurant, i.e., a wax or MG in different oils, are presented in Figure 2, while the micrographs of the oleogels composed of the combination of waxes and MGs at equal ratios are shown in Figure 3. Generally, dark zones represent oil, while the bright zones represent the crystal particles in the images of polarized microscopy.

#### 2.2.1. Polarized Light Micrographs of MG Oleogels

**Figure 4:**
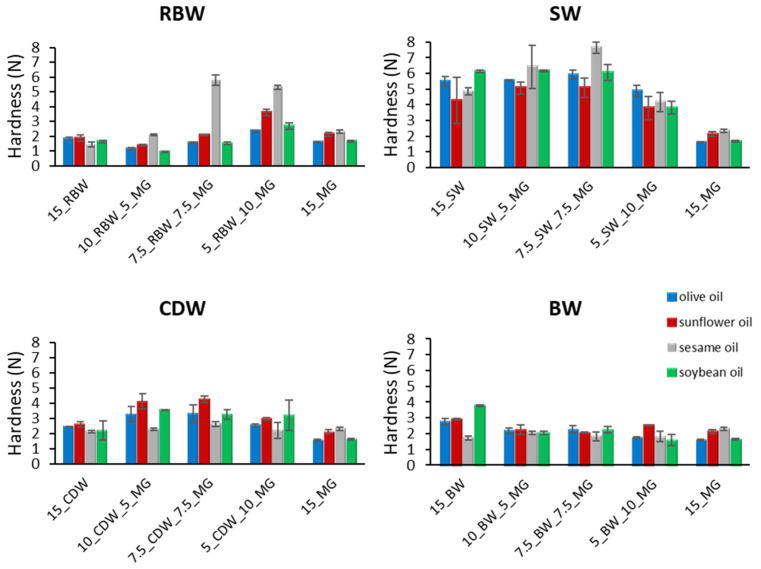
Hardness of oleogels created in different edible oils using monoglycerides (MGs) combined with waxes, namely rice bran wax (RBW), candelilla wax (CDW), sunflower wax (SW), and beeswax (BW) (mean value ± standard deviation).

The microstructure analysis of the pure MG oleogels showed bright needle-like crystals that were evenly distributed in all different oils, which is characteristic of MG crystallization. MGs are self-organized into an inverse lamellar phase with a β-subcell packing in oil, as it has been reported in the literature [40]. The morphology of the crystal structure did not change among the different edible oils; however, the size of the crystals varied. Specifically, larger crystals were present in soybean oil (average crystal length ~91 μm), intermediate-size crystals were present in sunflower oil (~58 μm) and olive oil (~54 μm), while smaller crystals were observed in sesame oil (~43 μm). The edible oils utilized for the oleogels have different compositions in terms of major and minor compounds [13]. Generally, the oils differ in the fatty acid composition of TAGs, i.e., in the chain length and in the unsaturation degree, as well as in the polar/non-polar functional groups of the components that they contain [41]. More specifically, the primary fatty acid in olive oil is oleic acid (approximately 70%), while linoleic acid (approximately 15%), and palmitic acid (approximately 17%) are present at lower concentrations [42]. Sesame oil is mainly composed of oleic (~41.5%), linoleic (~40.5%), and palmitic (~12.5%) acids. Sunflower oil is composed of linoleic (~58%), oleic (~29%), and palmitic (~6.5%) acids, while soybean oil contains linoleic (~51%), oleic (~23%), palmitic (~10%), and alpha-linolenic (~7%) acids [43]. Based on the microscopy findings, the different fatty acid distributions of the edible oils led to different MG crystal sizes. The non-polar oils, namely soybean oil and sunflower oil, resulted in larger crystals in contrast to the more polar oils, namely olive oil and sesame oil, which showed smaller crystals. Additionally, the variation in the shape and size of the MG crystals in the oleogels showed a correlation with the texture characteristics of the samples, where smaller crystals resulted in harder gels, as discussed in Section 2.3 (Figure 4). Hence, these findings are consistent with previous studies that have demonstrated the impact of various oil properties, such as polarity, on the gelation and crystallization behavior of MG oleogels [44,45].

#### 2.2.2. Polarized Light Micrographs of Wax-Based Oleogels

The behavior of waxes in oil gelling can be affected by their composition, as documented in previous studies [9]. In general, waxes are characterized as esters composed of long-chain fatty acids and fatty alcohols. Their oil gelling functionality is influenced by the specific compounds they contain, which can vary depending on their origin, including hydrocarbons, free fatty acids, free alcohols, etc. [46]. According to the literature, RBW is mainly composed of wax esters (92–97%) and free fatty acids (0–2%), while free fatty alcohols and hydrocarbons (n-alkane) are present in trace amounts [47]. Wijarnprecha et al. [6] report that RBW is mainly composed of wax esters (C44–C64), which comprise a mixture of saturated esters between fatty acids (mainly C22 and C24) and fatty alcohols (C24–C40) [6,47,48]. CDW contains a high proportion of hydrocarbons (50–65%, mainly C31), followed by wax esters (27–35%), free fatty acids (7–10%, C16–22), and free fatty alcohols (10–15%, mainly C30) [35,44]. Wax esters (96–97%, mainly C20–22) are the dominant components of SW, while it also contains a low proportion of free fatty acids (3%, C16–22); free fatty alcohols and hydrocarbons are present in trace amounts [5,25,47]. Lastly, BW mainly consists of wax esters (58–59%, C16, C24–32), followed by hydrocarbons (26–27%, C27–31), free fatty acids (8–9%, C16, C24), and free fatty alcohols (6–7%, C24–32) [25].

Concerning the oleogels structured with 15% (*w*/*w*) RBW, the crystals of RBW displayed an elongated needle-like shape and formed well-structured crystal matrices that can entrap significant amounts of oil [32]. Moreover, 15% (*w*/*w*) SW formed smaller needle-like crystals than RBW. In contrast, 15% (*w*/*w*) BW oleogels exhibited spherulites with very small sizes, while 15% (*w*/*w*) CDW oleogels displayed a homogeneous crystalline matrix with small crystals. It is well-established in the literature that the crystal morphology of waxes is greatly influenced by their chemical composition [25]. The formation of long, needle-like crystals has been attributed to the presence of high amounts of wax esters [49]. Despite having a wax ester content similar to SW, RBW showed a difference in crystal size, possibly due to the existence of minor compounds like free fatty acids and resins [25]. Fayaz et al. [40] also reported similar findings on the crystal size and morphology in RBW and SW oleogels. In contrast, CDW and BW had a considerably lower wax ester content (22–35% and 58–59%, respectively), and their different crystal morphology could be attributed to the existence of free fatty acids, free fatty alcohols, and hydrocarbons [25]. Other researchers have also observed spherulites or spherical agglomerates in the case of BW [36,50], and it has been concluded that needle-like crystals are formed at low concentrations of BW, whereas spherulites are created at higher concentrations [50]. In contrast, CDW oleogels formed small crystals with a very homogenous crystalline structure, which is consistent with the findings of Winkler-Moser et al. [36], who reported a uniform dispersion of randomly oriented platelets with an average length scale of about 5 μm.

No differences were observed in the crystal morphology of wax oleogels formed with different edible oils. These results are in agreement with Dassanayake et al. [48] who reported similar crystals in RBW oleogels formed with olive oil and salad oil (mixed canola oil and soybean oil, 50:50).

#### 2.2.3. Polarized Light Micrographs of Mixed Oleogels

Different crystalline structures were observed in oleogels formed by a combination of waxes and MGs in edible oils. Generally, the crystalline structure seems to be significantly influenced both by the interaction of the gelators and by the composition of the oils used for the oleogel formation. Specifically, in the case of RBW with olive oil, the length of the needle-like crystals decreased as the percentage of RBW decreased. A similar trend was noted in the case of sunflower oil, except for the sample with a 5% RBW concentration, where the formation of spherulites was observed. On the contrary, using sesame oil for oleogelation the formation of spherulites was much more pronounced at a concentration of 5% RBW, while spherulites combined with small crystals were observed in the 7.5% RBW–7.5% MG oleogels. Lastly, in the case of soybean oil, spherulites and Maltese crosses appeared (Figure 3), indicating the presence of anisotropic, birefringent structures. Generally, as the ratio of RBW to MG increased, fewer spherulite crystals developed, signifying the important role of RBW in the crystalline structure of the oleogels, as also reported by Chen et al. [51].

Regarding the CDW oleogels, the CDW crystals were much smaller than the MG needle-like crystals; therefore, as the MG concentration increased in the system, the size and density of the crystals also increased in the case of olive oil and sunflower oil. Concerning soybean oil oleogels, spherulites appeared at a concentration level of 7.5% CDW–7.5% MG (Figure 3). A similar structure, but of denser and smaller crystals, was observed in the case of 5% CDW–10% MG. In addition, spherulites were observed in sesame oil oleogels (Figure 3), while small and sparsely dispersed crystals also appeared in the case of 10% CDW–5% MG sesame oil oleogels. Da Silva et al. [22] suggest that the relative concentration of each component is the main driving factor for the crystal morphology, rather than the overall concentration of the structurants. In the CDW oleogels, very small crystals appeared; however, when CDW was combined with MGs, spherulites formed. This is probably due to the interaction of hydrocarbons (n-alkane) present in CDW with MGs, resulting in the formation of spherulites.

In the case of olive, sunflower, and soybean oil oleogels with mixtures of SW and MGs, the morphology of the crystals was needle-like; however, the length of crystals was much smaller than those formed by each structuring agent separately (MG and SW). On the contrary, when sesame oil was used, spherulites were observed at mixtures of 7.5% SW–7.5% MG, while this type of crystals appeared to be denser and larger in size in the case of the combination of 10% SW–5% MG. Similar results were observed by Barroso et al. [23], who studied the combination of SW and MGs at a total concentration of 6% *w*/*w* in flaxseed oil. The authors observed that the variation in the proportion of MG and SW components in the mixture had a significant impact on the crystal morphology. They proposed that the polar MG groups interacted with the polar groups of fatty acids and fatty alcohols in SW, leading to an improvement in the crystal structure and therefore an increased affinity of the oleogelators.

The crystal structures formed by BW were very small compared to the MG crystals. The crystal morphology was modified by adding MGs to the systems, resulting in a combination of spherulitic crystals with small needle-like crystals in the background, for all oils, except for soybean oil. The formation of spherulites was more intense as the concentration of MGs increased in the system (5% BW–10% MG). In the case of olive oil, spherulite crystals appeared and their size increased as the percentage of MGs increased, creating fewer but larger crystals. Similar behavior was observed in the case of sunflower oil, while in sesame oil oleogels, both large spherulite crystals and smaller needle-like crystals were present, suggesting that needle-like crystals were probably MGs. On the contrary, in the case of soybean oil, in all combinations of BW and MG, small-sized needle-like or plate-like crystals were observed.

The interaction between the waxes and MGs led to the formation of crystals with different morphologies. It should be noted that although the crystallization conditions, such as temperature and cooling rate, were constant, the crystal morphology varied depending on the oil and structurants used. The composition of the liquid oil phase can influence the microstructure of the oleogels by affecting the compatibility between the wax and oil phases.

### 2.3. Textural Properties

#### 2.3.1. Single-Component Oleogels

Some interesting patterns were observed regarding the hardness of the oleogels. The statistical analysis performed for the single-component oleogels (15% W, 15% MG) showed that SW oleogels were significantly harder, followed by BW and CDW. On the other hand, MG and RBW were softer compared to the others. All waxes, except for RBW, resulted in harder oleogels compared to MG oleogels (Figure 4). RBW created the softest gels probably because a higher concentration of RBW (>10% *w*/*w*) is required to form a solid stable gel [52]. The differences in the hardness of oleogels are probably due to the composition of the wax esters and hydrocarbons, which are mostly responsible for gel formation. Furthermore, based on previous literature reports, there is a positive association between the gel strength of wax-based oleogels and the chain length of the wax esters, as well as the concentration of free fatty acids. Specifically, a higher proportion of longer chain fatty acids in the wax ester and a greater quantity of free fatty acids results in a stronger gel, as reported by Fayaz et al. [40]. On the other hand, MGs formed larger crystals than waxes, as shown in the microscopy images, and created softer gels since larger crystal size is associated with lower gel strength and firmness [53]. Therefore, the presence of more cohesive networks with higher amounts of small fat crystals contributes to harder texture compared to the samples with fewer and larger crystals [22].

All oleogels produced in this study were significantly harder compared to traditional animal fat products, namely butter (from cow’s milk) and pork lard. Specifically, the hardness value of butter and lard measured at 25 °C was 0.22 N and 0.19 N, respectively. In relation to the wax-based oleogels, the average hardness for different oils was 5.18 N for SW, 2.80 N for BW, 2.38 N for CDW, and 1.71 N for RBW, while the average value of hardness for MGs was 1.91 N. Previous studies [18,19] have shown that MG oleogels, either alone or in combination with phytosterols at a concentration level of 15% (*w*/*w*), are stable and can be successfully used to replace animal fat in meat products [15]. Regarding the waxes, the minimum concentration for SW to form stable gels in several edible oils was 1.0–1.5% (*w*/*w*) [52]. A similar gelling behavior was reported for CDW in safflower oil (0.5–1.0 % *w*/*w*) [30] and rice bran oil (1.0–1.5 % *w*/*w*) [46], while BW was an efficient oleogelator in forming strong gels at a concentration level of 1.0–1.5% (*w*/*w*) in olive oil [54] and 1.5–2.0% (*w*/*w*) in rice bran oil [46]. On the other hand, RBW required concentration greater than 8–10% (*w*/*w*) to form a self-standing oleogel in several oils [52]. According to Frolova et al. [26], the hardness of oleogels created by 6% *w*/*w* BW in olive, sunflower, and linseed oils ranged from 1.17 to 1.63 N, indicating stable gels. Moreover, Winkler-Moser et al. [36] measured the hardness of SW, CDW, and BW oleogels in soybean oil at a concentration level of 5% *w*/*w* and found that the hardness was 1.6, 2.5, and 0.8 N, respectively. Da Silva et al. [22] studied the combination of CDW, MG, and fully hydrogenated crambe oil (HF) at low concentration (5–10% *w*/*w*) in soybean oil and high oleic sunflower oil and found that the hardness of oleogels ranged from 0.1 to 0.85 N. Thus, the results of the present study indicate that the oleogels formed were shelf-stable and hard enough to replace animal fat in food products. Regarding the different edible oils used to form wax-based oleogels, sunflower oil and soybean oil created the harder gels (Figure 4), while olive oil formed medium-hardness gels, and sesame oil formed the softest oleogels. On the contrary, in the case of MG-based oleogels, sesame oil produced the hardest gels. These results indicate that the polar compounds of olive oil and sesame oil can affect the crystal structure, yielding softer gels in the case of waxes. The microstructure and hardness of the oleogel can also be influenced by the composition of the liquid oil phase, as it can affect the compatibility between the wax and oil phases. According to Borriello et al. [55], oils containing a higher proportion of saturated fatty acids were found to promote the development of a more tightly packed structure in carnauba wax oleogels.

#### 2.3.2. RBW and MG Oleogels

In the case of the mixtures of RBW and MG oleogels, the addition of MGs in concentrations up to 7.5% *w*/*w* led to softer gels compared to the single-component gels, except for sesame oil oleogels (Figure 4). On the contrary, the 5% RBW and 10% MG mixtures produced significantly harder gels for all oils (*p* < 0.05). According to our preliminary experiments and in agreement with Holey et al. [52], the minimum concentration of RBW to form a gel was 10%. Thus, the samples containing 15% RBW were softer than MG oleogels, and the addition of low concentrations of MGs (<7.5% *w*/*w*) did not significantly affect the oleogels’ hardness. In contrast, when a small amount of wax was added to the MGs (5% RBW and 10% MG), a synergistic effect between the components was observed, resulting in significantly harder gels compared to those made of the individual components. These results can be attributed to the different crystal formations, as observed from the microscope images (spherulites and Maltese crosses). Therefore, oleogels with a higher amount of spherulites were harder. Thus the 5% RBW and 10% MG mixtures formed harder gels compared to the other combinations. Regarding the use of different oils to form the RBW and MG gels, sesame oil formed much harder gels, followed by sunflower oil, and olive oil and soybean oil had the lowest hardness (Figure 4). It is worth noting that the spherulite crystal formation was observed more extensively in the concentration of 5% RBW and 10% MG, and specifically in sesame oil, which is related to the increased hardness of the gels. Sesame oil seems to have very good compatibility with the combination of RBW and MGs probably due to minor components it contains such as polyphenols, specifically sesamin and sesamolin, which are present only in this type of edible oil.

#### 2.3.3. BW and MG Oleogels

Mixtures of BW and MG oleogels did not result in significantly harder gels than individual MGs and those formed by the other waxes. According to the results, the hardest gels were formed by pure wax for all oils, apart from the case of sesame oil, indicating an incompatibility between the two components. The hardness of oleogels containing mixed structural agents did not exhibit significant variations when different oils were used, except for the oleogels containing 5% BW and 10% MG in sunflower oil, which had higher hardness (Figure 4).

#### 2.3.4. CDW and MG Oleogels

In the case of the mixtures of CDW and MG oleogels, a trend was observed regarding the hardness (Figure 4), which followed the order 7.5% CDW, 10% CDW, 15% CDW, and 5% CDW in all oils used. Synergistic interactions between the components were observed, and they resulted in higher oleogel hardness as the wax concentration increased in the gel. The differences in gel hardness were found to be associated with the changes in the crystal microstructure that were identified through microscopy. The interaction between other mixed gelator systems, such as different waxes, affecting the crystal microstructure and therefore the firmness of the system, were also reported in the literature [36]. On the contrary, da Silva et al. [22] studied the combination of CDW, MG, and HF (at a total concentration of 5–10% *w*/*w*) as structurants in soybean oil and high oleic sunflower oil and found that increasing amounts of MG or HF did not result in harder materials, even if the total concentration of structuring agents increased. Regarding the use of different oils, sunflower oil resulted in the hardest gels, followed by soybean oil and olive oil, and finally sesame oil. According to the microscope images, the formation of spherulites was much more pronounced in sesame, followed by soybean, olive, and sunflower oils in descending order, indicating that the more crystals appeared, the softer the oleogel was. These results are in contrast to those observed for RBW, suggesting that each wax behaves differently in structuring gels.

#### 2.3.5. SW and MG Oleogels

The SW combined with MG formed the hardest gels compared to all other treatments. As in the case of CDW, the order of hardness in terms of wax concentration in the mixtures was 7.5% SW, 10% SW, 15% SW, and 5% SW, while all the oleogels formed with wax combinations were much harder than the MG oleogels. It is worth noting that the formed oleogels exhibited minimal elasticity, breaking into two pieces during compression, a behavior which was slightly more pronounced in sesame oil. These results indicate that a lower concentration of wax and MG could probably be used to produce hard gels. Concerning the use of the different oils in the mixtures of SW and MG oleogels, sesame oil and olive oil resulted in the hardest gels.

Overall, the hardest gels were produced by SW, CDW, RBW, BW, and MG in de-creasing order (Table 1). In relation to the use of different oils, sunflower oil and sesame oil resulted in the hardest gels, followed by soybean oil and olive oil (Table 1). Furthermore, MG addition in the wax-based oleogels had a different impact on texture, depending on the wax and oil type, suggesting a different three-way interaction between the MGs, the wax components, and the oil triglycerides that lead to different crystalline structures. The combination of 7.5% W and 7.5% MG exhibited the highest hardness, while the 15% MG oleogels had the lowest (Table 1).

### 2.4. Melting Point

**Figure 5:**
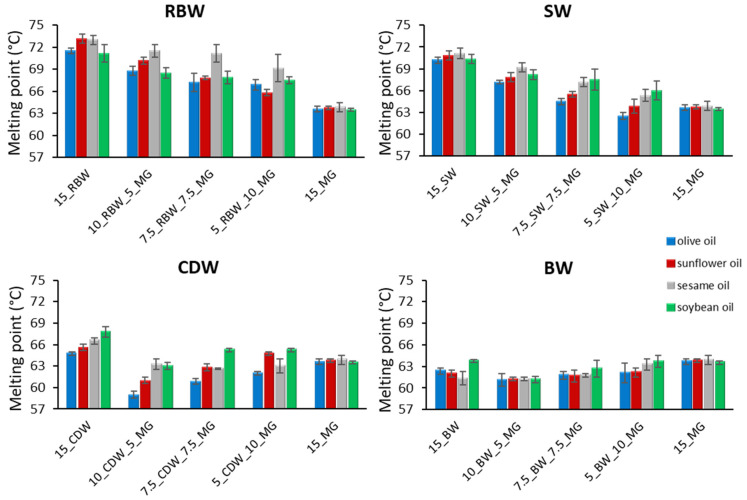
Melting points of oleogels created in different edible oils using monoglycerides (MGs) in combination with waxes, namely rice bran wax (RBW), candelilla wax (CDW), sunflower wax (SW), and beeswax (BW) (mean value ± standard deviation).

The melting point of the single-component oleogels with different waxes was 71–73 °C, 70–71 °C, 65–68 °C, and 61–62.5 °C, for the RBW, SW, CDW, and BW, respectively, while the melting point of MG oleogels was 63–64 °C for the oils examined (Figure 5). The gels had lower melting points compared to those of the solid waxes and MGs. Waxes behaved differently depending on the oil that was used to form the gels. In the case of RBW and SW, sunflower oil and sesame oil oleogels had a higher melting point than olive oil and soybean oil oleogels. However, the opposite behavior was observed for BW oleogels, while CDW in soybean oil had the highest melting point. These results are in agreement with Winkler-Moser et al. [36] who examined the melting points of oleogels with DSC, and they found that SW, CDW, and BW had melting points at 75.7, 65, and 63.7 °C, respectively. Although there were differences observed among the treatments, statistical analysis did not indicate any significant differences among the oils employed (Table 1).

Regarding the combinations of waxes with MGs, in the case of RBW and SW, it was observed that with increasing concentrations of MGs, the melting point of the oleogels decreased (Figure 5). This is expected because MGs have a considerably lower melting point compared to RBW and SW. However, in the case of BW, which had a lower melting point than that of MG, increasing concentrations of MG resulted in a slight increase in the melting point of the oleogels. An interesting behavior occurred in the case of CDW, as the addition of a small amount of MG decreased the melting point of the gels compared to that of pure wax. This probably occurred due to the different crystal structures formed, as observed in the microscope, with the effect of lowering the melting point. Regarding the different oils, soybean oil and sesame oil lead to slightly higher melting points, followed by sunflower oil and olive oil. These differences, however, were not statistically significant (Table 1).

### 2.5. FTIR Measurements

**Figure 6:**
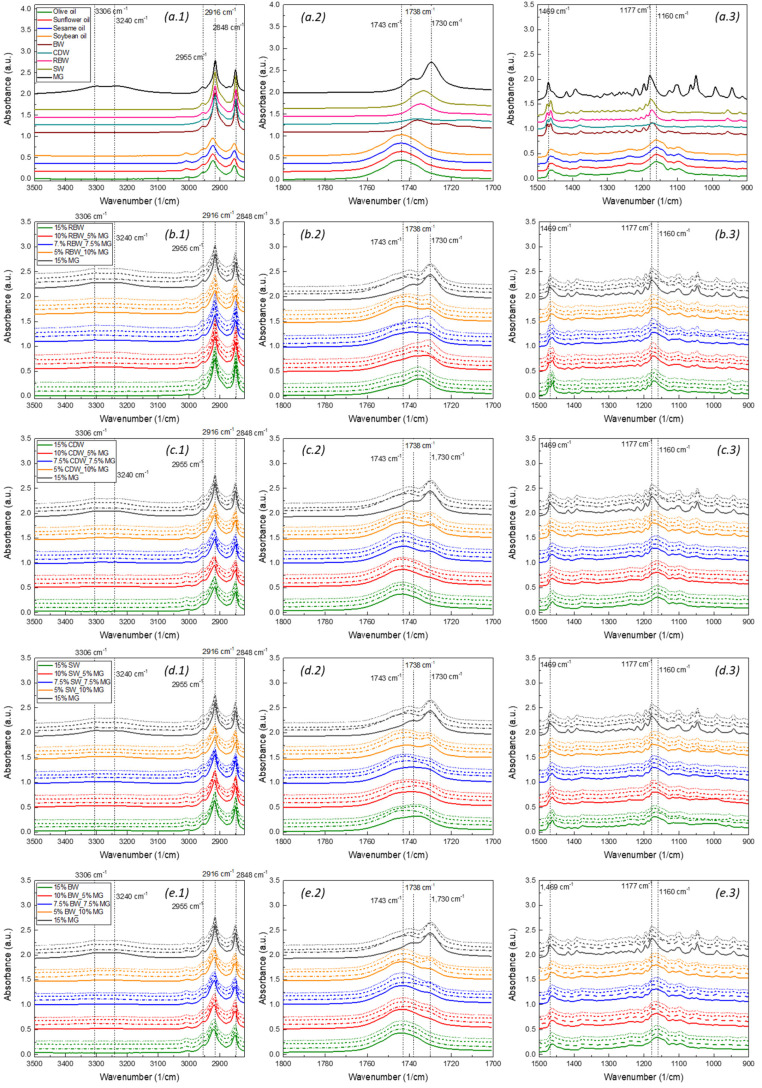
FTIR spectra of (**a.1**–**a.3**) pure structurants and oils; (**b.1**–**b.3**) oleogels prepared with monoglycerides (MGs) and rice bran wax (RBW); (**c.1**–**c.3**) oleogels prepared with MGs and candelilla wax (CDW); (**d.1**–**d.3**) oleogels prepared with MGs and sunflower wax (SW); and (**e.1**–**e.3**) oleogels prepared with MGs and beeswax (BW), in different edible oils (solid lines: olive oil; dashed-dotted lines: sesame oil; dashed lines: soybean oil; and dotted lines: sunflower oil). FTIR spectra of (**a.1**–**a.3**) pure structurants and oils; (**b.1**–**b.3**) oleogels prepared with monoglycerides (MGs) and rice bran wax (RBW); (**c.1**–**c.3**) oleogels prepared with MGs and candelilla wax (CDW); (**d.1**–**d.3**) oleogels prepared with MGs and sunflower wax (SW); and (**e.1**–**e.3**) oleogels prepared with MGs and beeswax (BW), in different edible oils (solid lines: olive oil; dashed-dotted lines: sesame oil; dashed lines: soybean oil; and dotted lines: sunflower oil).

FTIR measurements were used to investigate the interactions among the components of the oleogels, including the structural agents and liquid edible oils. The spectra of the oleogels with RBW, CDW, SW, and BW exhibited slight variations, which suggest differences in the chemical and structural characteristics of the gels. The FTIR spectra of all oils, waxes, and oleogels are presented in Figure 6.

Oleogels with MGs in all edible oils showed two peaks in the 3200–3350 cm^−1^ wavenumber range, at 3306 and 3240 cm^−1^ (Figure 6(b.1)), which correspond to intermolecular hydrogen bonding between the (3-OH) alcohol and the carbonyl ester (C=O) groups of MGs [18,24]. In contrast to MG oleogels, oils, waxes, and wax oleogels (Figure 6(a.1)) did not show these peaks, as these hydrogen bonds were not formed. In the mixed systems with waxes and MGs, these peaks became more distinct, as the concentration of MGs increased (Figure 6(b.1,c.1,d.1,e.1)). It is noted that in all sesame oil oleogels, only the peak at 3306 cm^−1^ appeared. Öğütcü and Yılmaz [56] observed similar results, where they reported that weak intermolecular -OH hydrogen bonds existed only in MG oleogels due to the presence of hydroxyl groups, while no -OH hydrogen bonding was observed in carnauba wax oleogels. Similarly, Yılmaz and Öğütcü [57] found no -OH hydrogen bonding in beeswax or beeswax oleogels.

The spectral range from 3000 to 2800 cm^−1^ corresponds to the medium-strength C–H alkane bond. RBW, CDW, SW, and BW (Figure 6(b.1,c.1,d.1,e.1)) showed peaks at 2954, 2916, and 2848 cm^−1^, while MG oleogels showed peaks at 2955, 2914, and 2849 cm^−1^, indicating the presence of cis- single-bond C–H stretching [58]. The intensity of the peaks was higher in RBW and SW compared to CDW and BW, as waxes contain a long-chain alkane backbone [59]. The absorption peak at 1743 cm^−1^, corresponding to the stretching vibration of carbonyl group C=O, was observed in all liquid oils, as shown in Figure 6(a.2). Regarding the oleogels with waxes, RBW showed an absorption peak at 1735 cm^−1^, with both CDW and BW at 1735 cm^−1^ in all used oils, and SW at 1736 cm^−1^ in olive, sunflower, and soybean oils, while at 1741 cm^−1^ in sesame oil (Figure 6(b.2,c.2,d.2,e.2)). On the other hand, in MG oleogels, two peaks appeared at 1738 and 1730 cm^−1^ in olive oil, 1739 and 1730 cm^−1^ in sunflower oil and soybean oil, and 1740 and 1731 cm^−1^ in sesame oil, which had a different absorbance intensity. According to Modupalli et al. [58], these peaks can be associated with a strong asymmetric C=O stretch bonds of vinyl or phenyl, α, β-unsaturated ester linkages, and carboxylic acid entities. In the oleogels created by combinations of waxes and MGs, these peaks were located between the peaks that appeared in the single-component oleogels (waxes or MGs) and were proportional to the concentration of the components used. There were some slight differences regarding the absorbance intensity of these peaks depending on the oil used to form the gels in some cases.

Furthermore, differences in the spectra among waxes and MGs were observed in the region of 1475–1460 cm^−1^, resulting in peaks that were also present in the oleogels and were proportional to the concentration of the components contained in the mixed oleogels. These peaks correspond to the CH_2_ bending and scissoring vibration of the acyl chains of lipids [60]. Finally, small differences in the spectra of the oleogels were observed in the range of 1400–1000 cm^−1^ (Figure 6(b.3,c.3,d.3,e.3)), which mainly correspond to the C–O stretching vibration in the ester group [60] and C–H bending vibrations [18].

Overall, the FTIR results suggest that the studied oleogels are structured based on physical interactions of their components that lead to the formation of a dense crystalline network that entraps and immobilizes the liquid oil, without the formation of new hydrogen or covalent bonds.

### 2.6. Mixed-Component Oleogels in Foods As a Fat Substitute

The oleogels formed by a combination of waxes and MGs in edible oils could be used as a fat substitute in a plethora of foods, such as meat products (fermented sausages, emulsion-type sausages, burgers, meat patties, etc.), dairy products (ice cream, cheeses, etc.), confectionery products (cookies, cakes, bread, etc.), chocolate, and spreadable products (margarine, fillings, creams, toppings, mayonnaise) [61]. Depending on the food product in which fats are used, they are responsible for specific desired attributes, namely structure, texture, flavor, and other specific properties [62]. Specifically, oleogels structured with SW combined with MGs could be used in meat products because they were very hard and therefore may simulate pork backfat functionality in these products. In the case of RBW and MG oleogels, the gels formed were hard and had the highest melting points, which probably makes them suitable for confectionery products, which are heated in an oven. Lastly, oleogels with CDW or BW and MGs had sufficient hardness and a smooth surface, characteristics which would be suitable for dairy products, such as ice cream. Thus, wax-MG-based oleogels can be used as potential healthy replacements of animal fat (saturated and *trans* fats) in several products, reducing the calorie intake and meeting consumer demand for healthier products.

## 3. Conclusions

The current investigation has demonstrated that the composition and characteristics of the combined structurant oleogels are influenced by the type of the waxes, the concentration of the wax and the monoglycerides present in the system, and the oil used. The hardest structure was produced by SW followed by CDW, RBW, BW, and MG. Concerning the structurant concentrations, 7.5% W and 7.5% MG created oleogels with the highest hardness, while the MG oleogels had the lowest. In addition, the study demonstrated that the crystalline structures varied depending on the type of wax, its concentration in the oleogels, and the oil utilized, with the MG oleogels exhibiting the largest crystal structures and the CDW oleogels the smallest. The RBW oleogels had the highest melting point, followed by SW, CDW, MG, and BW in descending order. In addition, higher melting points were observed in the case of soybean oil and sesame oil, followed by sunflower oil and olive oil. The obtained data can be utilized to modify the desired properties of oleogels for a number of potential food applications. Specifically, the harder gels, such as SW and CDW gels, could be used as pork backfat substitutes in meat products, whereas the softer structures, such as RBW or BW with MGs, could be used as milk fat substitutes in dairy products. Further research is required to deeply comprehend the interplay between the constituents and the impact of minor components present in the waxes and oils on the crystallization of the system.

## 4. Materials and Methods

### 4.1. Raw Materials

Rice bran wax (RBW), candelilla wax (CDW), and sunflower wax (SW) (Daraveli and Co., Ltd., Athens, Greece) and beeswax (BW) (white pharma grade, Syndesmos S.A., Athens, Greece) were used in the present study in combination with monoglycerides (MGs) (HARI 95 Riketa SDN BHD, Johor Bahru, Malaysia) for the formation of different oleogels. According to the manufacturer’s specifications, the MGs had a minimum monoester content of 95%, a maximum acid value of 3%, a maximum iodine value of 2%, and a maximum free glycerine value of 1%. Based on the manufacturer’s specifications, RBW had an acid value of 7 mg KOH/g and a saponification value of 80 mg KOH/g, while CDW had an acid value of 18 mg KOH/g, and a saponification value of 61 mg KOH/g. Furthermore, SW had an acid value of 2.3 mg KOH/g and a saponification value of 91.6 mg KOH/g, while BW had an acid value of 20 mg KOH/g and a saponification value of 95.5 mg KOH/g. According to the manufacturers’ specifications, the melting point of the studied structuring agents was 79 °C, 75 °C, 71.5 °C, and 64 °C, for RBW, SW, CDW, and BW, respectively, whereas MG had a melting point of 71 °C.

Four different edible oils, namely, olive oil (Minerva S.A., Metamorphosi, Greece), sunflower oil (Minerva S.A., Metamorphosi, Greece), sesame oil (Haitoglou Bros S.A., Kalochori, Greece), and soybean oil (Marata S.A., Athens, Greece), were purchased from the local market. These edible oils were chosen due to their different fatty acid composition and polar components, as well as their widespread usage in the food industry. It is worth noting that olive oil and sesame oil are considered high-nutritional value oils, offering documented health benefits (REFS).

### 4.2. Oleogel Preparation

To form the oleogels, the concentration of the gelators was maintained at a constant level of 15% *w*/*w*, while the precise mass ratios of the waxes/MGs were varied as presented in Table 2. The concentration level of 15% *w*/*w* of waxes or MGs was chosen based on previous studies [18,19], as this concentration level resulted in the formation of solid and hard gels that could be utilized in food applications. Additionally, 3 levels of wax-MG combinations were selected in order to evaluate the interaction between the different components on the structural properties of the gel. Even though the waxes have different gelling ability (defined as the lowest concentration at which they form a gel), the same concentration levels were used for all studied waxes, so that the results could be comparable. A full factorial experimental design was applied, where all wax-oil-MG combinations were prepared, giving a total of 68 different formulations.

To prepare the oleogels, the edible oils were first heated to 85 °C with continuous stirring, and then the structuring agents were slowly added (Table 2) and thoroughly mixed until completely dissolved [18,19]. Following the addition of the structuring agents, the mixtures were stirred for 30 min at a temperature range of 90–95 °C. Next, the liquified oleogels were placed into cylindrical containers (27.7 mm diameter) and glass tubes (10 mm diameter) and cooled at room temperature for 40 min. The oleogel samples were stored at a temperature of 5 °C for 24 h. All measurements were taken at room temperature after equilibrating the oleogel samples at 25 °C for 3 h. The measurements were performed twice using different batches of oils.

### 4.3. Color Measurement

The oleogel color determination was carried out by measuring the parameters L* (lightness), a* (+/−, red to green), and b* (+/−, yellow to blue) using a Chroma Meter CR-400 (Minolta, Osaka, Japan) with a D 65 illuminant [19]. Five measurements were obtained for each oleogel sample. The results were expressed as mean ± standard deviation.

### 4.4. Optical Microscopy

Polarized light microscopy has been widely used to observe the microstructure of the gels and to understand the morphology of the crystals formed. The microstructure of the oleogels was analyzed by polarized light microscopy, following the method described by Moschakis et al. [63]. To perform this analysis, a drop of liquified oleogel sample was placed on a preheated microscope slide, and a glass cover slip was placed over it. The samples then followed the procedure described above for cooling, storage conditions, and equilibration before measurements. The microstructural images were acquired using a ×10 magnification objective in an Olympus BX43 polarizing microscope (Olympus Optical Co., Ltd., Tokyo, Japan) equipped with a microscope digital camera (Basler USB3 Vision, Ahrensburg, Germany). Four images of each sample were recorded using Basler Microscopy Software (v.2.1) (Basler, Ahrensburg, Germany).

### 4.5. Texture Analysis

The textural properties of the oleogels were assessed using the penetration test with a Universal TA.XT plus Texture Analyzer (Stable Micro Systems, Godalming, Surrey, UK) equipped with a 5-kg load cell. Oleogels were shelf-stable and hard enough to cut with a shear blade. For the measurements, oleogels were cut into cylindrical samples of 10 mm height and 27.7 mm diameter. A P6 stainless-steel cylindrical probe (6 mm in diameter) was used for the penetration test. The penetration speed was set at 0.5 mm/s, and the penetration depth was 5 mm, which corresponds to 50% of the height of the samples [18,22]. Two parameters were measured to evaluate the texture properties: hardness, which is defined as the maximum peak force for the penetration; and gel strength, which is the total area under the force–time curve. Five measurements were performed for each treatment, and the results are presented as mean ± standard deviation.

### 4.6. Melting Point Determination

The determination of the melting point of the oleogels was carried out following the method described by Zampouni et al. [18]. In brief, aliquots of molten oleogels (7 g) were transferred into glass tubes (10 mm diameter), and the same storage procedure was followed as in the rest of the analyses. The glass tubes were placed in a water bath at 38 °C and were heated at a constant rate of 1 °C/3 min until the samples were totally melted and were converted into a transparent liquid. During the heating process, two temperature readings were recorded: the softening point (when the sample began to soften) and the clearing point (when the sample became entirely clear and liquid-like). The melting point of the oleogels was reported as the average of the two temperature readings. Each treatment was measured in triplicate, and the results were expressed as mean ± standard deviation.

### 4.7. Fourier-Transform Infrared Spectroscopy (FTIR)

The chemical structure of the oleogels was analyzed using a Fourier-transform infrared spectrometer (FTIR 6700 series, JASCO, Tokyo, Japan) equipped with 3-reflection ATR diamond (MIRacle ATR, Pike Technologies, Madison, Wisconsin, U.S.). The oleogel samples, after equilibration for 3 h at 25 °C, were placed onto the ATR plate and pressed with a swivel tip for adequate contact. The spectra were recorded over the wavenumber range of 4000 to 500 cm^−1^, with a spectral resolution of 4 cm^−1^, and 32 scans were collected at room temperature [18]. Before each measurement, a background air spectrum was scanned and subtracted from the spectra of each sample. The ATR crystal was cleaned with isopropanol and dried before the next sample was analyzed. Five spectra were obtained for each treatment, and the average spectrum was used for interpretation. The spectra were analyzed using Spectra Manager (V.2, Jasco, Tokyo, Japan).

### 4.8. Statistical Analysis

The data presented in this study were reported as the mean values and standard deviation of two fully replicated experiments. The full-factorial model statistical analysis results are presented in Table 1 for each main factor (e.g., structurant concentration, wax concentration, and edible oil type). In addition, separate statistical analyses were performed for each wax and oil type, in order to assess more thoroughly the effect of the interactions of the different variables (interactions between MGs and waxes, oils and waxes, etc.). The statistical analysis of the data was carried out using ANOVA with the general linear model, and the level of significance was set at 0.05. Differences among treatments were identified using Tukey’s test. The statistical software used for the analysis was MINITAB v.16 (Minitab, Inc., State College, PA, USA).

## Figures and Tables

**Table 1:** Effect of structural agent type, concentration, and oil, on physicochemical parameters of oleogels.

Factors	Physicochemical Parameters			Color Parameters		
	Hardness (N)	Gel Strength	Melting Point (°C)	L*	a*	b*
Structural agent *						
MGs	1.91 ^c^	13.97 ^d^	64.53 ^c^	55.1 ^d^	−4.4 ^a,b^	7.1 ^d^
RBW	2.34 ^b,c^	16.95 ^c,d^	69.23 ^a^	69.4 ^b^	−4.3 ^a^	12.3 ^b^
CDW	2.87 ^b^	22.25 ^b^	63.59 ^c^	63.0 ^c^	−5.1 ^c^	13.9 ^a^
SW	5.49 ^a^	43.04 ^a^	67.32 ^b^	74.1 ^a^	−4.5 ^a,b^	10.5 ^c^
BW	2.24 ^c^	19.21 ^b,c^	61.55 ^d^	63.3 ^c^	−4.8 ^b,c^	7.9 ^d^
*SE*	0.08	0.66	0.29	0.21	0.4	0.1
*p*-value	<0.001	<0.001	<0.001	<0.001	<0.001	<0.001
*Concentration of wax*						
0.0%	1.91 ^c^	13.97 ^c^	64.53 ^b^	55.06 ^d^	−4.4 ^a^	7.1 ^e^
5.0%	3.09 ^b^	25.23 ^b^	64.21 ^b^	64.34 ^c^	−4.7 ^b^	9.0 ^d^
7.5%	3.63 ^a^	28.40 ^a^	64.73 ^b^	67.30 ^b^	−4.8 ^b^	10.2 ^c^
10.0%	3.06 ^b^	23.62 ^b^	64.91 ^b^	68.85 ^a^	−4.8 ^b^	11.3 ^b^
15.0%	3.16 ^b^	24.20 ^b^	67.83 ^a^	69.34 ^a^	−4.4 ^a^	14.2 ^a^
*SE*	0.09	0.74	0.32	0.24	0.4	0.1
*p*-value	<0.001	<0.001	<0.001	<0.001	<0.001	<0.001
*Edible oil*						
Olive oil	2.79 ^b^	22.49 ^a,b^	65.26 ^a^	65.58 ^b^	−6.7 ^c^	16.1 ^a^
Sunflower oil	3.14 ^a^	24.91 ^a^	65.34 ^a^	63.60 ^c^	−3.5 ^a^	5.7 ^c^
Sesame oil	3.13 ^a^	22.48 ^b^	65.32 ^a^	67.04 ^a^	−4.9 ^b^	13.9 ^b^
Soybean oil	2.82 ^b^	22.47 ^b^	65.05 ^a^	63.70 ^c^	−3.4 ^a^	5.8 ^c^
*SE*	0.08	0.66	0.29	0.21	0.4	0.1
*p*-value	<0.001	0.020	0.886	<0.001	<0.001	<0.001

* MGs: monoglycerides; RBW: rice bran wax; CDW: candelilla wax; SW: sunflower wax; BW: beeswax; SE: standard error. ^a–e^ Means with the same letters at the same column within each factor are not significantly different (*p* < 0.05).

**Table 2:** Composition of the experimental formulations of wax (W)—monoglyceride (MGs) oleogels.

Samples ^1^	Wax (% *w*/*w*)	MG (% *w*/*w*)	Oil (% *w*/*w*) ^2^
15_W	15.0	0.0	85.0
10_W_5_MGs	10.0	5.0	85.0
7.5_W_7.5_MGs	7.5	7.5	85.0
5_W_10_MGs	5.0	10.0	85.0
15_MGs	0.0	15.0	85.0

^1^ W denotes different waxes, namely beeswax, candelilla wax, rice bran wax, and sunflower wax. ^2^ Oil corresponds to different edible oils, i.e., olive, sunflower, sesame, or soybean oil.

## Data Availability

The data presented in this study are available on request from the corresponding author.
